# Surgical Treatment of Rare Multiple Cardiac Myxomas: A 13-Year Clinical Experience at a Single Institution

**DOI:** 10.21470/1678-9741-2025-0145

**Published:** 2026-09-08

**Authors:** Wenjie Diao, Shengqiang Zhang, Shaofeng Yang, Yu Shi, Chongwen Shen, Chão Cai, Shicheng Xie, Chao Shi, Ge Liu

**Affiliations:** 1 Department of Cardiac Surgery, The First Affiliated Hospital of Bengbu Medical University, Bengbu, Anhui, People's Republic of China; 2 Graduate School of Bengbu Medical University, Bengbu Medical University, Bengbu, Anhui, People's Republic of China

**Keywords:** Surgical Intervention, Multiple, Recurrence

## Abstract

**Introduction:**

Cardiac myxoma (CM) is the most common primary cardiac tumor. Multiple CMs are rare and can lead to heart failure and arterial embolism. This study aimed to explore clinical characteristics, surgical treatment experience, and follow-up strategies for multiple CMs.

**Methods:**

A retrospective analysis was conducted on eight patients with multiple CMs who underwent surgical treatment. Clinical characteristics were analyzed by extracting relevant clinical data, electrocardiography and echocardiography findings, surgical treatment details, and postoperative follow-up information. All patients had undergone cardiopulmonary bypass surgery. Pathological analysis was performed on all tumors, and postoperative cardiac ultrasound follow-up was also conducted.

**Results:**

Multiple CMs were uncommon, representing only 5.9% of all cases. Among 136 patients with cardiac tumors admitted during the same period, only eight had multiple CMs. All patients with multiple CMs underwent complete tumor resection. The size of the resected tumors was approximately 5–80 (39.3 ± 20.3) mm. The follow-up period ranged from one to nine years. The recurrence rate was 37.5% (3/8), and all recurrent cases underwent reoperation for tumor resection. There were no perioperative deaths, and overall surgical outcomes were satisfactory.

**Conclusion:**

Our study reveals that surgery is the best strategy for multiple CMs. The tumor should be completely resected, and color Doppler echocardiography is recommended every six months postoperatively to detect tumor recurrence. Those cases of multiple myxomas have had high recurrence rate after resection (37.5%) but were submitted successfully to a new resection. Timely surgical resection provides favorable outcomes for patients with multiple or recurrent CMs.

## INTRODUCTION

Primary cardiac tumors are rare, with cardiac myxoma (CM) being the most common type, accounting for approximately 0.3% of all primary cardiac neoplasms^[1,2]^. CMs typically develop in the atria and consist of an acid mucopolysaccharide–protein matrix rich in polygonal stromal cells^[3]^. Most CMs are solitary tumors, while multifocal myxomas or those originating from different cardiac chambers are rarely observed. The clinical manifestations of multiple CMs vary widely and may include obstructive cardiac dysfunction, arterial embolism, or constitutional symptoms, all of which pose significant diagnostic and therapeutic challenges^[4]^. Due to the limited number of reported cases of multiple CMs, most studies are presented as individual case reports. Only a few small case series have described their diagnosis and treatment, and standardized treatment guidelines have yet to be established. This study presents our experience with eight patients diagnosed with primary multiple CMs who underwent surgical treatment at our center.The aim is to provide clinical insights into the diagnosis, surgical management, and follow-up strategies for this rare condition.

## METHODS

### Study Population

We retrospectively analyzed all patients with primary cardiac tumors who underwent surgical treatment at our department between 2012 and March 2025. All patients provided written informed consent for inclusion in the study, and the study protocol was approved by the Medical Ethics Committee of the Bengbu Medical University First Affiliated Hospital (2024KY012).

The mean age and weight, as well as the clinical presentations, were extracted from the clinical records. Findings from chest computed tomography (CT), electrocardiography (ECG), transthoracic echocardiography (TTE), and abdominal color ultrasonography were recorded. Data on tumor markers were also obtained. The mean cardiac parameters evaluated were as follows: left ventricular diameter (LVD), left atrial diameter (LAD), right ventricular diameter (RVD), right atrial diameter (RAD), left ventricular ejection fraction (LVEF), and fractional shortening (FS).

### Surgical Techniques

For all patients, surgical procedures, including left atrial myxoma resection via thoracoscopy, median sternotomy, and cardiopulmonary bypass (CPB), were performed using superior and inferior vena cava cannulations. All procedures were performed under combined intravenous anesthesia with tracheal intubation. Surgical procedures included cold cardioplegia. Gentle manipulation was performed to avoid tumor fragmentation and embolism. The key surgical steps included: (1) tumor excision: wide resection of the tumor base to prevent recurrence; the oval fossa was repaired using a Dacron® patch; (2) cavity inspection: meticulous exploration of all four cardiac chambers to ensure complete removal of multifocal or residual tumors; (3) valve assessment: mitral and tricuspid valve functions were evaluated, and repairs were performed if necessary. A postoperative TTE was performed before discharge.

### Statistical Analyses

In this retrospective study, descriptive statistics measures were used, including mean value, standard deviation, median, percentiles, and percentages.

## RESULTS

### Patient Characteristics

We retrospectively analyzed 136 patients with primary cardiac tumors who underwent surgical treatment. Of these, eight patients (5.9%) were diagnosed with primary multiple CMs, including one male and seven female patients (Table 1).

**Table 1:** Clinical characteristics of patients.

Variable	N (%)/Mean ± SD
Sex (male/female)	
Female	7 (87.5%)
Male	1 (12.5%)
Age, years (mean ± SD)	41 ±15.3
Weight (kg)	61.2 ± 6.8
NYHA class	
NYHA I	1 (12.5%)
NYHA II	4 (50%)
NYHA III	2 (25%)
NYHA IV	1 (12.5%)
Symptoms	
Cerebral infarction	1 (12.5%)
Right lower limb embolism	1 (12.5%)
Physical examination	2 (25%)
Cough, chest tightness	4 (50%)
Electrocardiogram	
Sinus tachycardia	2 (25%)
T wave change	1 (12.5%)
Normal	5 (62.5%)
Cardiac parameters	
Left ventricular diameter (mm)	47.1 ± 4.1
Left atrial diameter (mm)	34.9 ± 5.7
Right ventricular diameter (mm)	23.6 ± 4.5
Right atrial diameter (mm)	42.8 ± 9.3
Ejection fraction (%)	57 ± 5.7
Fractional shortening (%)	29.6 ± 4.1
Dimension of tumor (mm)	39.3 ± 20.3

NYHA=New York Heart Association; SD=standard deviation

The mean age of the patients was 41 ± 15.3 years (range: 15 – 65 years), with a mean weight of 61.2 ± 6.8 kg (range: 52 – 72 kg). Clinical presentations included chest tightness, cough, lower-limb edema, lower-limb arterial embolism, and acute cerebral infarction. All eight patients underwent chest CT, ECG, TTE, and abdominal color ultrasonography. Tumor markers were assessed in four patients. The mean cardiac parameters were as follows: LVD, 47.1 ± 4.1 mm; LAD, 34.9 ± 5.7 mm; RVD, 23.6 ± 4.5 mm; RAD, 42.8 ± 9.3 mm; LVEF, 57 ± 5.7%; FS, 29.6 ± 4.1 %. ECG revealed arrhythmia (sinus tachycardia) in two patients. Echocardiography revealed hemodynamic disturbances in five patients, with two patients presenting evidence of tricuspid regurgitation and mild pulmonary hypertension.

### Surgical Outcomes and Pathology Findings

One patient underwent left atrial myxoma resection via thoracoscopy, whereas the remaining seven patients underwent median sternotomy. CPB was performed using superior and inferior vena cava cannulations. Mild hypothermia was maintained during CPB, with a bypass duration of 38 – 202 (106.5 ± 51.9) minutes and aortic cross-clamping time of 19 – 126 (63.1 ± 36.1) minutes. Surgical procedures included intermittent cold blood cardioplegia in seven patients and cold crystalloid cardioplegia in one. Gentle manipulation was performed to avoid tumor fragmentation and embolism.

Pathological examination confirmed myxomas in all eight patients. Tumor locations were as follows: one patient had two right atrial myxomas (Table 2); one had tumors in the left atrium (LA) and left ventricle (LV) (Table 2, case 3, Figure 1); one had tumors in the LA and right ventricle (RV) (Table 2, case 6, Figure 2); two patients each had two myxomas in the LA; two had tumors in the LA and right atrium (RA); and one patient had tumors in the RA, RV, and LA. The tumor size ranged from 5 to 80 mm (mean: 39.3 ± 20.3 mm). The New York Heart Association functional classification was Grade I in one patient, Grade II in four, Grade III in two, and Grade IV in one. Elevated cancer antigen 125 (CA-125) levels were observed in three patients. One patient (Table 2, case 5, Figure 3) was diagnosed with a lower-limb arterial embolism, with thrombi revealing CM. No perioperative deaths occurred. One patient experienced postoperative injury (electrical damage to the lower extremity nerves), whereas the remaining patients recovered well. The follow-up period ranged from one to nine years.

**Table 2:** Location and size data of primary multiple cardiac myxomas.

Case number	Tumor location and tumor size (mm)				Carney complex	CA-125 (lU/ml)	Recurrent
	RA	RV	LA	LV			
1	-	-	50 x 25, 28 x 17	-	No	NC	No
2	10x5x5	-	30 x 30 x 20, 10x10x5	-	No	NC	Yes
3	-	-	50 x 40 x 40	30 x 30 x 20	Yes	178.5	Yes
4	-	-	50 x 40 x 40, 40 x 30 x 20	-	No	3.84	No
5	40, 20, 60	-	40 x 20	-	No	NC	No
6	-	40 x 20 x 20	60 x 50 x 50	-	Yes	423.9	Yes
7*	80 x 60, 70 x 60	-	-	-	No	219	No
8	22 x 25 x 25	27 x 38 x 42	17 x 23 x 17	-	Yes	NC	No

*The kidney tumor ruptured 13 months later

CA125=cancer antigen-125 (normal range = 0-35 Ill/ml); LA=left atrium; LV=left ventricle; NC=not checked; RA=right atrium; RV=right ventricle

**Fig. 1:**
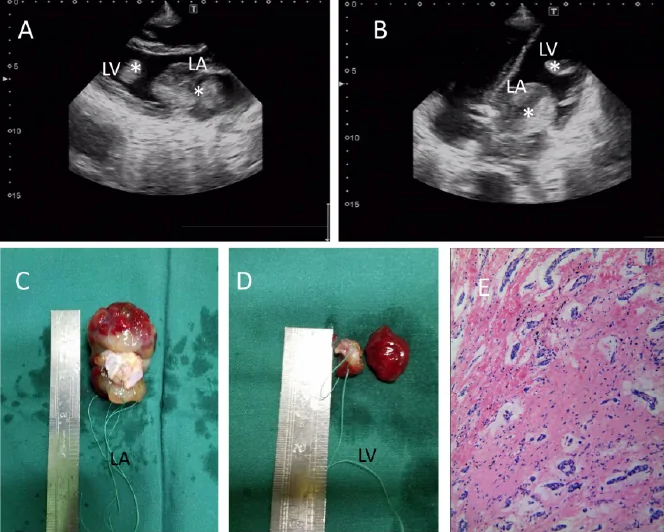
Case 3: left atrium (LA) and left ventricle (LV) myxomas. A, B) Echocardiographic images. C, D) Surgical resection of two tumors (one from the LA and the other from LV). E) Histopathological findings of myxoma, hematoxylin, and eosin staining. *The tumors.

**Fig. 2:**
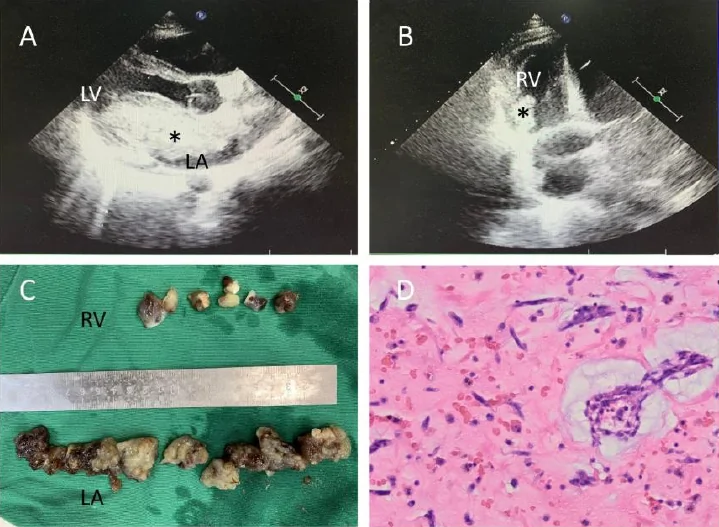
Case 6: left atrium (LA) and right ventricle (RV) myxomas. A, B) Echocardiographic images. C) Surgical resection of two tumors (one from LA and the other from RV), formalin-soaked myxoma. D) Histopathological finding of myxoma, hematoxylin, and eosin staining. *The tumors.

**Fig. 3:**
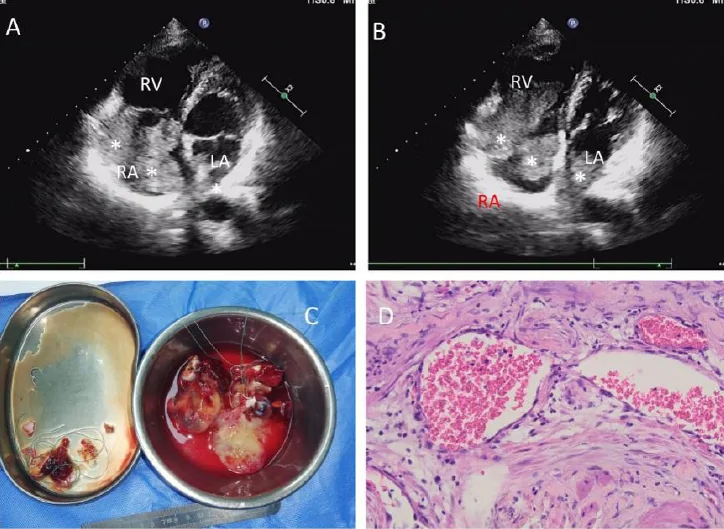
Case 5: left atrium (LA) and right atrium (RA) myxomas. A, B) Echocardiographic images. C) Surgical resection of four tumors (three from the LA and the other from the RA). D) Histopathological finding of myxoma, hematoxylin, and eosin staining. *The tumors.

Three cases of recurrence were observed. Case 2 involved a patient who had undergone left atrial single myxoma resection five years earlier. Case 3 (Figure 1) had a history of leg skin myxoma surgery and CM consistent with the Carney complex, which recurred 34 months later, presenting as tumors measuring 41 x 49 mm in the RA, 30 x 45 mm in the LA, and 11 x 19 mm in the LV, accompanied by mild pulmonary hypertension. The patient underwent complete tumor resection at a high-level hospital; however, recurrence within the heart was reported during a recent telephone follow-up. Cases 6 (Figure 2) and 8, representing the same patient, experienced recurrence in three heart chambers four years after surgery. Genetic analysis revealed a protein kinase cAMP-dependent type I regulatory subunit alpha (PRKAR1A) mutation consistent with the Carney complex.

## DISCUSSION

Multiple CMs are rare. We identified eight patients in our cohort with primary multiple CMs, accounting for 5.9%o (8/136) of patients with cardiac tumors admitted during the same period. Multiple CMs rarely originate from different cardiac regions. In our group, multiple tumors were confined to a single cardiac chamber, whereas others were distributed across multiple chambers. The maximum number of tumors was identified in one patient with myxoma involving three cardiac chambers. Goel et al.^[5]^ reported four tumors in three chambers, including two ventricles, and Satish et al.^[6]^ described sporadic CM involving all four cardiac chambers. Previous studies have reported familial and Carney syndrome-associated cases of CMs. In addition, multiple primary elastic fibromas, lymphomas, and other cardiac tumors have been reported^[7^ ^8^ ^9]^. Because of the limited available research, the true incidence of multiple CMs remains uncertain.

Early diagnosis of CMs is difficult, as most asymptomatic or nonspecific symptoms cannot be identified in a timely manner. Although the clinical manifestations of multiple myxomas are similar to those of single CMs, they tend to be more severe. Intracardiac obstruction and peripheral embolism are the two main dinical manifestations in patients with cardiac tumors. Earlier reports identified embolization in approximately 30 – 40% of cases^[10]^. Intracardiac blood flow obstruction caused by cardiac tumors was the most common clinical finding, occurring in 60 – 70% of cases and representing the main reason for patient referral^[11]^.

In our cohort, one case was identified during a routine examination, and five cases were associated with different degrees of heart failure, accounting for 44%o of the group. Larger or more numerous tumors were associated with more severe symptoms. Arterial embolism occurred in two cases, including one with lower-extremity arterial embolism and one case with acute cerebral infarction. Although myxomas are histologically benign, they can cause severe complications or sudden death. Therefore, clinicians should remain vigilant to avoid unnecessary delays in diagnosis. Preoperative ECG findings in patients with multiple primary cardiac tumors were nonspecific^[12]^.TTE is prioritized because it is noninvasive, convenient, and cost-effective^[13]^. Although TTE has high sensitivity, it may fail to determine whether a heart cavity contains several lesions, especially in cases with multiple tumors. Among the 20 lesions identified in eight cases in our cohort, one case involved three myxomas in the RA, and one lesion was missed. Additional examination methods, including cardiac CT, cardiac magnetic resonance imaging, and three-dimensional reconstruction techniques, are the key imaging modes used in this procedure, allowing surgeons to visually assess the location, size, and pedicle of the tumor before surgery^[14^ ^15^ ^16]^.These modalities facilitate complete tumor removal and help minimize perioperative risks. Myxoma cases in this group were confirmed using echocardiography and histopathology. Multiple CMs require differential diagnosis from other cardiac tumors, such as hemangiomas, sarcomas, and metastatic lesions.

Surgery reduces the risk of vascular embolic events and associated complications due to tumor detachment. Once diagnosed, myxomas should be treated promptly. The choice of surgical incisions should be based on the affected heart chambers, tumor size, number, and the attachment sites. Nass et al.^[17]^ reported that while tumors occur in both the LA and RV, the ones in the RV are missed on color ultrasound, requiring a second operation. In this study, each heart chamber was carefully explored to avoid intraoperative omissions. All four chambers were examined through a right atrio-atrial septal incision because most myxomas were centrally located rather than near the cardiac apex. This procedure facilitated the removal of tumors in both ventricles through the atrioventricular valve opening. A left atrial or ventricular incision may be considered when complete resection is difficult; however, ventricular incisions carry great risks and require careful myocardial protection. Before completing the intracardiac procedure, all four heart chambers and pulmonary vein openings were carefully examined to ensure that no residual or fragmented tumor tissues remained. The tumors were confirmed to be intact, and the heart chambers were repeatedly rinsed with a large amount of normal saline. For large tumors, the operation can be more difficult, as the tumors involve a wider area and are not easy to resect; thus, heart transplantation may be considered. Previous studies have reported that large heart tumors can be successfully resected using heart transplantation^[18,19]^.

The recurrence rate of multiple myxomas has rarely been reported. Previous studies have shown that the recurrence rate of atrial myxoma ranges from 5% to 14%^[20^ ^21^ ^22]^. In this study, the recurrence rate during one to 12 years of follow-up was 2.2% (3/136). However, the recurrence rate among patients with multiple myxomas was 37.5% (3/8), which was much higher than that reported for common CMs. Some scholars believe that recurrence is primarily related to the brittleness and multiple characteristics of the tumor. It is also suggested that recurrence may also be related to insufficient surgical resection or intraoperative implantation of tumor cells caused by surgery^[23,24]^. Patients with a family history of tumors or chromosomal abnormalities (*i.e.,* aneuploidy) may also have a higher recurrence risk^[25]^. For patients with multiple CMs, a family history of single myxomas, or suspected Carney syndrome, screening for the PRKAR1A gene mutation may help identify patients who are at high risk of recurrence.

In addition, there were three patients in this group with high CA-125 levels, more than five-fold higher than the normal value, and two of them were recurrence cases. This finding suggests that patients with elevated CA-125 should be closely monitored for possible recurrence. However, no published studies have yet reported a correlation between CA-125 and multiple myxomas, and further research is needed.

Previous surgical treatment strategies and our own clinical experience with multiple CMs strongly suggest that patients require close follow-up involving assessment of all cardiac chambers. Thus, it is best to perform the color Doppler ultrasound of the heart every six months. Once recurrence is identified, prompt reoperation is warranted to prevent severe complications caused by tumor embolization or excessive growth that could complicate resection.

### Limitations

This study involved standard medical procedures performed on only eight patients. The rarity of this disease, the small sample size, difficulties in controlling confounding factors, the amplification of random errors, and the lack of diversity verification all limit the interpretability and generalizability of the results. Future studies should include more patients with primary multiple cardiac tumors, use larger cohorts, and combine data from multiple institutions to allow systematic analyses that support early diagnosis and help identify patients at higher risk of recurrence.

## CONCLUSION

For primary CMs, surgery remains the best treatment strategy. Surgical procedures should be thorough, and patients should undergo echocardiography every six months after surgery to monitor for recurrence. Early surgical resection leads to better outcomes for CMs, including recurrent cases. Those cases of multiple myxomas have had high recurrence rate after resection (37.5%) but were submitted successfully to a new resection. Future studies should also collect more cases and data to analyze the correlations among gene mutations, tumor markers, multiple occurrences, and recurrence.

## Data Availability

The authors declare that the data supporting the findings of this study are available within the article.
